# Can bacterial lysates be useful in prevention of viral respiratory infections in childhood? The results of experimental OM-85 studies

**DOI:** 10.3389/fped.2022.1051079

**Published:** 2022-11-21

**Authors:** Stefania Ballarini, Ledit Ardusso, José Antonio Ortega Martell, Oliviero Sacco, Wojciech Feleszko, Giovanni A. Rossi

**Affiliations:** ^1^ Medicine and Surgery Department, University of Perugia, Perugia, Italy; ^2^ Allergy and Immunology Department, Rosario School of Medicine, National University of Rosario, Rosario, Argentina; ^3^ Health Sciences Institute, Autonomous University of Hidalgo State, Pachuca, Hidalgo, México; ^4^ Department of Pediatrics, Pulmonary and Allergy Disease Unit, G. Gaslini University Hospital, Genoa, Italy; ^5^ Department of Pediatric Pulmonology and Allergy, The Medical University Children's Hospital, Warszawa, Poland; ^6^ Department of Pediatrics, Unit of Pediatrics Pulmonology and Respiratory Endoscopy, G. Gaslini Hospital, Genoa, Italy

**Keywords:** respiratory infections, bacterial lysates, OM-85, HRV, RSV, IFV, SARS-CoV-2, trained immunity

## Abstract

Respiratory tract infections (RTI) are mainly viral in origin and among the leading cause of childhood morbidity globally. Associated wheezing illness and asthma are still a clear unmet medical need. Despite the continuous progress in understanding the processes involved in their pathogenesis, preventive measures and treatments failed to demonstrate any significant disease-modifying effect. However, in the last decades it was understood that early-life exposure to microbes, may reduce the risk of infectious and allergic disorders, increasing the immune response efficacy. These results suggested that treatment with bacterial lysates (BLs) acting on gut microbiota, could promote a heterologous immunomodulation useful in the prevention of recurrent RTIs and of wheezing inception and persistence. This hypothesis has been supported by clinical and experimental studies showing the reduction of RTI frequency and severity in childhood after oral BL prophylaxis and elucidating the involved mechanisms. OM-85 is the product whose anti-viral effects have been most extensively studied *in vitro*, animal, and human cell studies and in translational animal infection/disease models. The results of the latter studies, describing the potential immune training-based activities of such BL, leading to the protection against respiratory viruses, will be reported. In response to human rhinovirus, influenza virus, respiratory syncytial virus and severe acute respiratory coronavirus-2**,** OM-85 was effective in modulating the structure and the functions of a large numbers of airways epithelial and immune cells, when administered both orally and intranasally.

## Introduction

Respiratory tract infections (RTIs) are often associated with significant morbidity and, in childhood, represent one of the most common reasons of unscheduled medical visits and hospitalization (1, 2). Viruses are the predominant cause of such infections, but those of mixed etiology are not uncommon (3). However, RTIs often cause inappropriate antibiotic prescriptions, thus contributing to the development of antibiotic resistance, a major public health concern (3, 4). Infants and preschool children who experience severe respiratory virus infections, are at increased risk to develop airway hyperresponsiveness and eventually, loss of respiratory function later in life (5). Because of the paucity and not totally effective preventive measures and treatment, viral RTIs and their consequences such as wheezing illness and asthma, still represent an unmet medical need in children and other fragile populations (6–8). Over the last decades, attempts have been made to reduce the increased susceptibility to respiratory symptoms and morbidity, and epidemiological studies have demonstrated that living in a rural environment and some farming communities, may increase the efficiency of the immune responses against infectious pathogens and immune tolerance towards allergens (9–11). The evidence that early-life exposure to microbes, favoring the maturation and a healthy gut microbiota may have protective effects, has suggested that microbial-related products or metabolites such as prebiotics, probiotics, and bacterial lysates (BLs), could help prevent recurrent RTIs (rRTIs) and the onset of atopic disorders through “heterologous immunomodulation” of the body's natural defenses (9). These products may act on gut microbiota composition and indirectly exert immunomodulatory functions to provide an effective therapeutic option in patients with rRTIs (9–16). Among the several BLs available for clinical use, OM-85 is the product whose immunity training and anti-viral effects have been most extensively studied in *in vitro* animal and human cell studies and in translational animal infection/disease models (10, 17–19). The findings of the basic and applied studies designed to understand the biological mechanisms involved in those effects, will be summarized, and discussed in this review.

## OM-85: composition and mode of action on immune effector cells not exposed to viruses

OM-85 comprises the lyophilised fractions of 21 bacterial strains of the following respiratory pathogens: *Haemophilus influenzae*, *Streptococcus pneumoniae*, *Klebsiella pneumoniae* subspecies pneumoniae and ozaenae, *Staphylococcus aureus*, *Streptococcus pyogenes, Streptococcus sanguinis*, and *Moraxella catarrhalis* (17). This drug, which possesses immune stimulating and regulating/anti-inflammatory properties, is indicated for the prevention of rRTIs in adults and children above 6 months of age (17–21). The rationale to use BLs in rRTIs prevention is based on the hypothesis of their ability to mimic the natural exposure to germs, promoting the maturation and the re-organization not only of the gut but also of the lung microbiota, and favoring the immune homeostasis, (i.e., the crosstalk between gut microbiota and immune system), in the airways *via* the gut-lung axis (17–19). The bacterial derived molecules of OM-85 act both, as pathogen-associated molecular patterns (PAMPs) on innate immunity and as antigens on adaptive immunity, which, reacting with pattern recognition receptors (PRRs) and other antigen receptor on immune effector and structural cells, activate both innate and adaptive immunity (19–21). The resulting heterologous mucosal immune responses regulate the anti-viral activities of dendritic cells (DCs), macrophages, CD4^+^ helper and CD8^+^ cytotoxic T-cells, regulatory T-cells, B-cells, natural killer (NK) cells against invading pathogens (19–22). DCs are major players in both innate and adaptive responses against viruses. Studies performed *in vitro* and *ex vivo* in experimental animals demonstrate that BLs can induce DC maturation, increasing the production of anti-viral cytokines/chemokines and the expression of surface molecules involved in antigen presentation (23–25). Through interaction with PRR receptors and activation of NF-kB and MAPK dependent pathways, in DCs cultures, OM-85 triggered the production of type I IFN-*β*, IL-8, CCL2, CCL20 and IFN-α, molecules involved in early immune reaction against viral infections, and of IL-12, a potent inducer of Th1 type lymphocytes (23, 25). After exposure to OM-85, a dose-dependent increase in the expression of MHC II, CD40, and CD86 surface proteins was also detected, molecules required for antigen presentation to T-cells (23). In blood monocyte and bone marrow-derived macrophage cultures, OM-85 increased IL-1, IL-2, and TNF-α release and oxygen radical production to kill TNF-α sensitive targets (26, 27). TNF-α is a cytokine which is produced by activated macrophages and possesses strong antiviral activity against human influenza viruses (IFV) (28). In mice, treatment with OM-85 induced a polyclonal activation of B-cells with the production of IFV- and Respiratory Syncytial Virus (RSV)-specific IgA and IgG in serum and bronchoalveolar lavage (BAL) of animals not previously exposed to these viruses (23). These virus-specific IgA and IgG were functionally active and significantly inhibited both IFV and RSV replication *in vitro*. Therefore, OM-85 could train innate immune cells which would be ready to cover broad heterologous protection against infections and, at the same time they could enhance the adaptive immune cells to mount a response following an infection.

## Anti-viral activities of OM-85: *in vitro* and mice model results

Studies involving human epithelial cell cultures, i.e., primary bronchial epithelial cells (BECs) or epithelial cell lines, and human-relevant disease mice models were designed to evaluate the anti-viral activities of OM-85 against human rhinovirus (HRV), IFV, RSV, and Severe Acute Respiratory Syndrome-Coronavirus-2 (SARS-CoV-2).

### HRV infections

HRV is an RNA virus known to be the etiologic agent of more than 50% of upper RTIs in humans worldwide. This virus is a leading cause of bronchiolitis in infants and the most common agent associated with wheezing illness in children and adults (29). Like all respiratory viruses, HRV recognized airway epithelial cells as the main target for infection. The primary function of the airway's epithelium is to act as a mechanical defensive barrier, aiding the maintenance of normal airway function. However, airway epithelial cells can also be actively involved in initiating and orchestrating immune and inflammatory responses through the expression of various receptors and effector proteins on their surface and the release of chemokines and cytokines, which recruit and activate inflammatory and immune effector cells (30). The intercellular adhesion molecule-1 (ICAM-1) is the major receptor for HRV-A and HRV-B, and the Cadherin related family member 3 (*CDHR3*) protein for HRV-C. Cell receptor binding is necessary or HRV to gain entry into the cells by endocytosis, whilst the interaction with Toll Like Receptor (TLR)2 on airway epithelial cell membranes and TLR3 and TLR7 in the endosomes and Melanoma Differentiation-Associated Protein 5 (MDA5) and retinoic acid-inducible gene I (RIG-I) in the cytoplasm modulates the RV-induced innate immune responses (30). In response to HRV infection, airway epithelial cells express chemokines such as CXCL-1, CXCL-5, and CXCL-8, growth factors, such as amphiregulin, epithelial cell growth factor (EGF) and epiregulin and a variety of Th2 cytokines.

#### OM-85 inhibited HRV infection in primary human BECs

**Figure 1:**
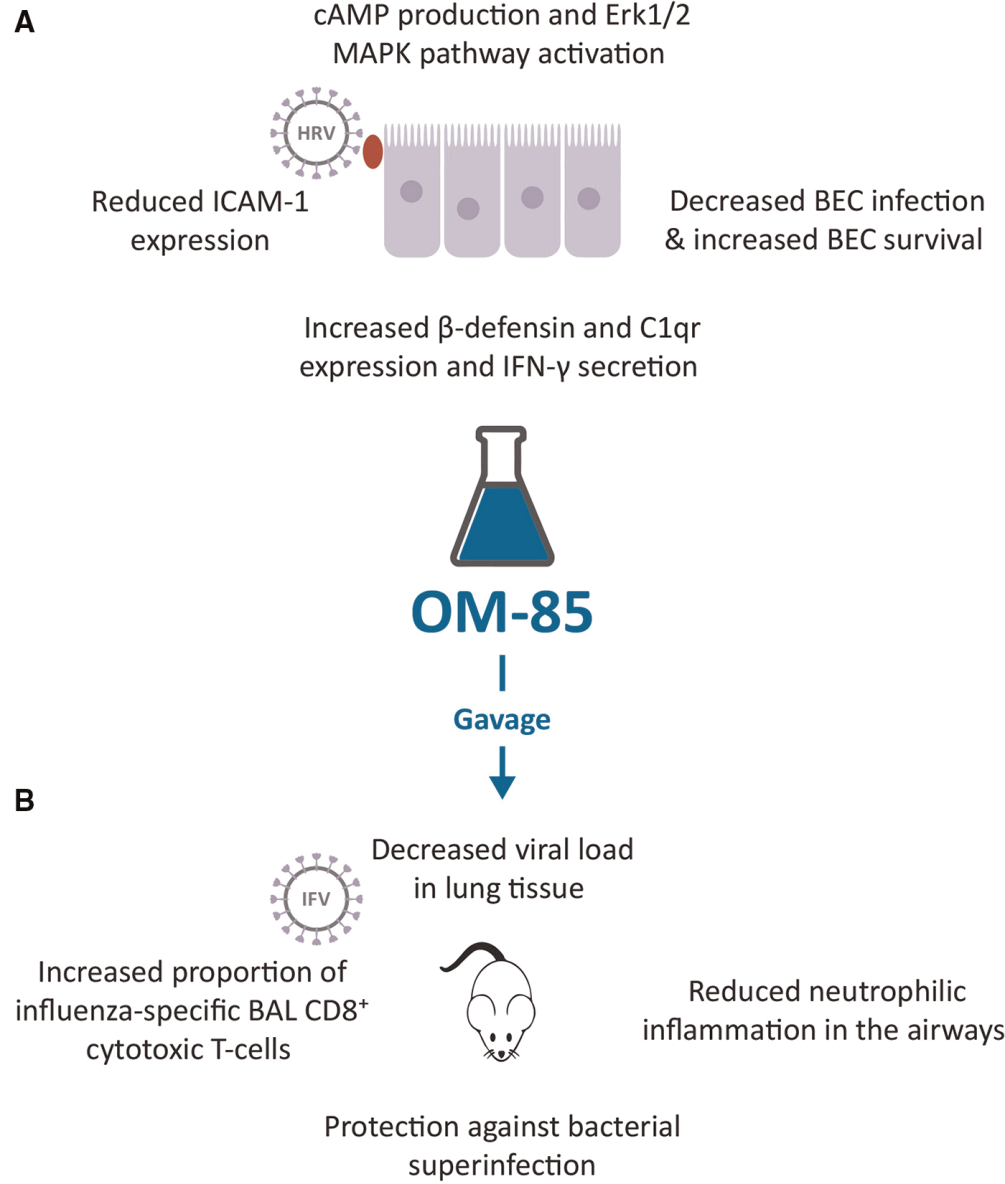
Inhibition of human rhinovirus (HRV) and influenza virus (IFV) infection by OM-85. (**A**). Effects of OM-85 on primary human bronchial epithelial cell (BEC) structural and functional changes, leading to decreased HRV infection and increased BEC survival. (**B**). Treatment of mice with OM-85 administered by gavage, decreased viral load in lung tissue, reduced neutrophilic inflammation in the airways, promoted the expansion of influenza-specific broncho alveolar lavage (BAL) fluid CD8^+^ cytotoxic T-cells and protected against bacterial superinfection, following IFV.

To determine whether OM-85 could interfere with HRV infection, a study using primary human BECs of otherwise healthy controls, asthma, and COPD patients was performed (Figure 1A) (31). The preventive effect of OM-85 pre-incubation (10 μg/ml) on HRV infection of BEC and on became significant at 24 and 48 h, was detectable at similar levels in all three cell donor groups and was associated with an increased cell survival at day 1, 2 and 3 (Figure 1A). When evaluating the signaling pathways involved, it was shown that OM-85 induced the activation of Erk1/2 MAPK, significant at 30 and 60 min, preceded by the formation of intracellular cAMP, significantly within 15 min, which subsequently declined. OM-85 also significantly increased the expression of virus interacting proteins *β*-defensin and complement component C1q receptor, two cell surface molecules known to bind HRV and to help to kill the virus intracellularly (32, 33). In addition, it significantly reduced the HRV-induced ICAM-1 expression, the main adhesion site for both HRV type A and B (34), in BECs of asthma and COPD patients (Figure 1A). Inhibition of cAMP signaling, and Erk1/ 2 MAPK pathway activation prevented most of the anti-viral activities of OM-85 in human BECs, showing that this OBL acted through these three signaling routes. Finally, pre-incubation with OM-85 further induced the secretion of anti-viral cytokine interferon (IFN)-*γ* significantly by BECs of all three patient groups. It therefore appears that OM-85 can enhance BECs' capacity to directly counter viral infections, by engaging a variety of cell functions. The possibility that these effects could be translated *in vivo*, is an attractive hypothesis.

### IFV infection

IFV is a major pathogen representing an ongoing health threat to several species, including humans. IFV infection, which is associated with a high risk of bacterial superinfection, is an important cause of respiratory morbidity and mortality in industrialized countries, despite the availability of anti-IFV specific vaccination (35). Upon infection with IFV the innate immunity plays a critical role in efficient and rapid control of viral infections, as well as in adaptive immunity initiation. The humoral immune system produces antibodies against different influenza antigens, whilst cell mediated immunity includes DC, CD4^+^ helper T cells and CD8 ^+ ^cytotoxic T cells. Both humoral and cell mediated responses are the arms of adaptive immunity (35).

#### OM-85 counteracted IFV infection in mice and bacterial superinfection

The possible OM-85 “anti-viral” activities against IFV infections, shown by the *in vitro* study (23), were confirmed in a mouse model. In mice, infected with sub-lethal doses of IFV-A, prior administration by gavage of 7.2 mg of OM-85-active principle for 10 days, induced a highly significant decrease in lung tissue viral load, observed at day 5 post infection (Figure 1B) (23, 36). At the same study time, a reduction of neutrophilic inflammation into the airways was detected in treated mice, because of the early control of the viral infection. This reduced neutrophilia was associated with an increase proportion of influenza nucleoprotein (NP)-tetramer^+^ specific CD8^+^ cytotoxic T-cell proportions in BAL fluid at day 10 post infection, indicating the activation of the adaptive immune response (23, 36). In the same experimental model, mice were exposed to a sublethal dose of either *Klebsiella pneumoniae* or *Streptococcus pneumoniae*, on day 7 following influenza infection (23, 36). Pre-treatment with OM-85 also protected mice from bacteremia, at 24 h post infection, and significantly reduced all morbidity signs, i.e., weight loss, body temperature increase, and disease score, at day 12 post infection (Figure 1B). Thus, the anti-influenza-specific activation of the immune responses induced by OM-85 pre-treatment, was also associated with an enhancement of some specific anti-bacterial responses. These findings support the concept of boosting anti-viral immunity, to increase alertness and clearance efficacy against secondary bacterial infections.

### RSV infection

RSV is a seasonal pathogen responsible for the highest percentage of severe bronchiolitis in pediatric patients but is also a major viral pathogen causing severe lung disease and exacerbations in the adult population, particularly among the elderly (37). The TLR4/CD14 complex is the main extracellular receptor recognizing RSV through binding with the F protein, while intracytoplasmic RIG-I, MDA5, and TLR3 PPRs recognize RSV transcripts and viral replication intermediates (29). The viral antigen recognition leads to nuclear factor-*κ*B activation with production IFN-*β*, a type-I IFN, which initiates the production of IFN-α, CXCL8, IFN-*λ* and TNF-α, by airway epithelial cells and innate immune cells (29). These cytokines recruit and activate polymorphonuclear leukocytes and natural killer cells and also trigger airway epithelial cells programmed death, a mechanism limiting viral spread to neighboring cells (29). Airway dendritic cells carry viral antigens to regional lymph nodes and where they activate CD4+ T-cells, CD8 + cytotoxic T-cells and B-lymphocytes which migrate back to the infected airways to kill the virus (29).

#### Reduction of RSV-induced viral load, weight loss, and lung inflammation in mice

**Figure 2:**
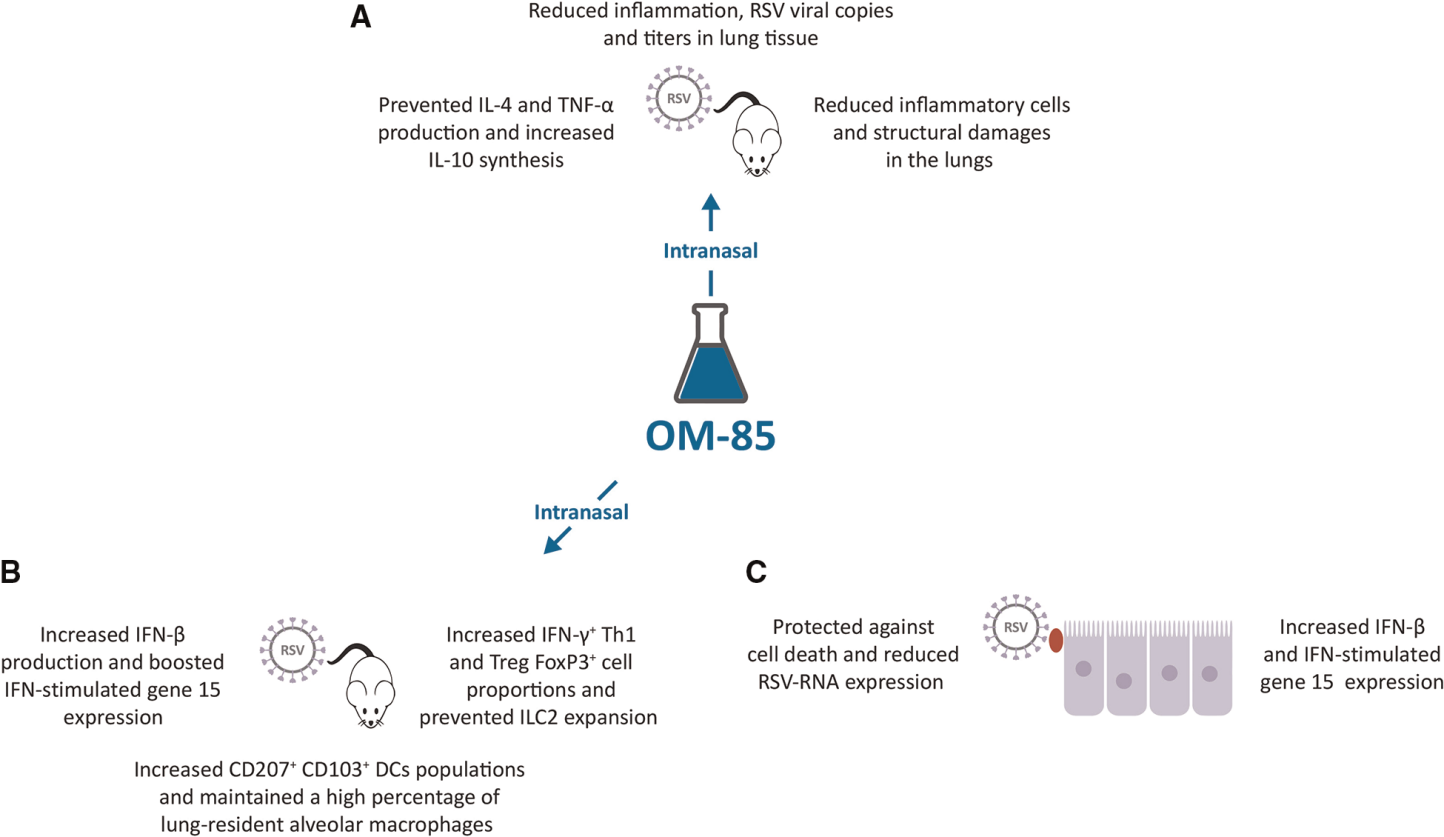
Inhibition of respiratory syncytial virus (RSV) infection by OM-85. (**A,B**). Intranasal (IN) treatment of Specific Pathogen Free (SPF) BALB/c mice with OM-85 reduced RSV infection in lung tissue promoting an effective innate and adaptive immune response. (**C**). Exposure of human airway cell line cultures to OM-85 protected against cell death and reduced RSV-RNA expression by increasing human interferon cytokine (IFN)-β and IFN-stimulated gene 15 expression.

With the background that an intranasal (IN) administration of microbiota-derived acetate was protective against RSV (38), and that OM-85 effectively inhibited HRV infection of BEC (31), a study was designed to investigate the potential protective effect of IN administration of OM-85 in RSV infected mice (39). IN OM-85 treatment (1 mg diluted in 20 ml/nostril of saline solution) was delivered to specific pathogen-free (SPF) BALB/c mice following four different schedules: (i) starting 12 h after infection and given daily throughout 5 days post infection or (ii) starting 1 day, (iii) or 3 days or (iv) 5 days prior to IN infection with RSV strain A2. OM-85 treatment administered 12 h after infection did not modify outcomes of established RSV infection i.e., lung viral load, weight loss and airway inflammation. OM-85 IN treatment given 1 day prior to infection and, on the same day, 6 h later, protected against weight loss on day 2 post-infection, maintained the animal weight slightly above compared to controls, but did not protect from lung perivascular and peri bronchial inflammation, despite a trend towards decrease in viral load. OM-85 IN treatment given 3 days prior to infection protected against weight loss at days 2 and 3 post-infection, significantly reduced viral load, decreased perivascular and peri bronchial inflammation, and reduced IL-4 production in the lung. Finally, OM-85 IN treatment given 5 days prior to infection reduced weight loss, cellular infiltrates into the lungs and viral expression and replication in the airways, peri-bronchial and perivascular inflammation scores (Figure 2A). Reduction in inflammatory cells and structural damages in the lungs, significantly prevented the production of IL-4 and TNF-α, while increasing the synthesis of the anti-inflammatory cytokine IL-10 (39).

#### Modulation of the lung immune response against RSV infection in mice

In these RSV-infected mice, IN treatment given 5 days prior to infection also significantly increased the proportion of the CD4^+^ IFN-*γ*^+^ Th1 cell and FoxP3^+^ Treg cell populations in the lung and prevented the RSV-induced increase of the innate lymphoid cell 2 (ILC2) proportions, cells known to be associated with Th2 type of immune response and with more severe RSV-bronchiolitis (Figure 2B) (39, 40). Moreover, the populations of tolerogenic CD207^+^ CD103^+^ DCs was increased in OM-85 pretreated RSV-infected mice, and in non-infected mice, thus suggesting a reprogramming of these DCs to promote less Th2 cell subtype activation (41). Finally, OM-85 treatment maintained a high percentage of lung-resident alveolar macrophages (AM), cells known to play an essential role in controlling RSV infection (42), increased the lung expression and production of the immunomodulator and IFN-*β*, and did boost the expression of the IFN-stimulated gene 15 (ISG15) (43–45). Finally, experiments were performed *in vitro* with human airway epithelial cells, a preeminent natural RSV target and important IFN-*β* producers (39). Pre-treated with OM-85 (7.5 mg/ml) protected A549 cell line cultures against cell death, reduced RSV-RNA expression in those cells, and increased IFN-*β* and ISG15 expression (Figure 2C). The protective role of OM-85 was abolished entirely in the absence of genes related to the type I IFN response, i.e., when A549 cells lacking the type I response pathway (IFNAR and RIG-I knockout cells) were used. Therefore, the broad protective effect of OM-85 against RSV infection is at least partially mediated through the modulation of the INF response.

### SARS-CoV-2 infection

COVID-19, the syndrome induced by SARS-CoV-2, is characterized by a wide spectrum of clinical manifestations ranging from a mild, self-limiting flu-like respiratory illness to life-threatening multiorgan failure and death (46). The severity of the clinical manifestation of SARS-CoV-2 infection is related to the viral virulence and load, but also to efficiency of the innate and adaptive immune response (47). The experimental results of recent publications from three independent groups suggest thar OM-85 could inhibit SARS-CoV-2 and a murine coronavirus infection and promote early innate immune training against the virus.

#### Downregulation of SARS-CoV-2 ACE2 receptor and TMPRSS2 transcription and expression

**Figure 3:**
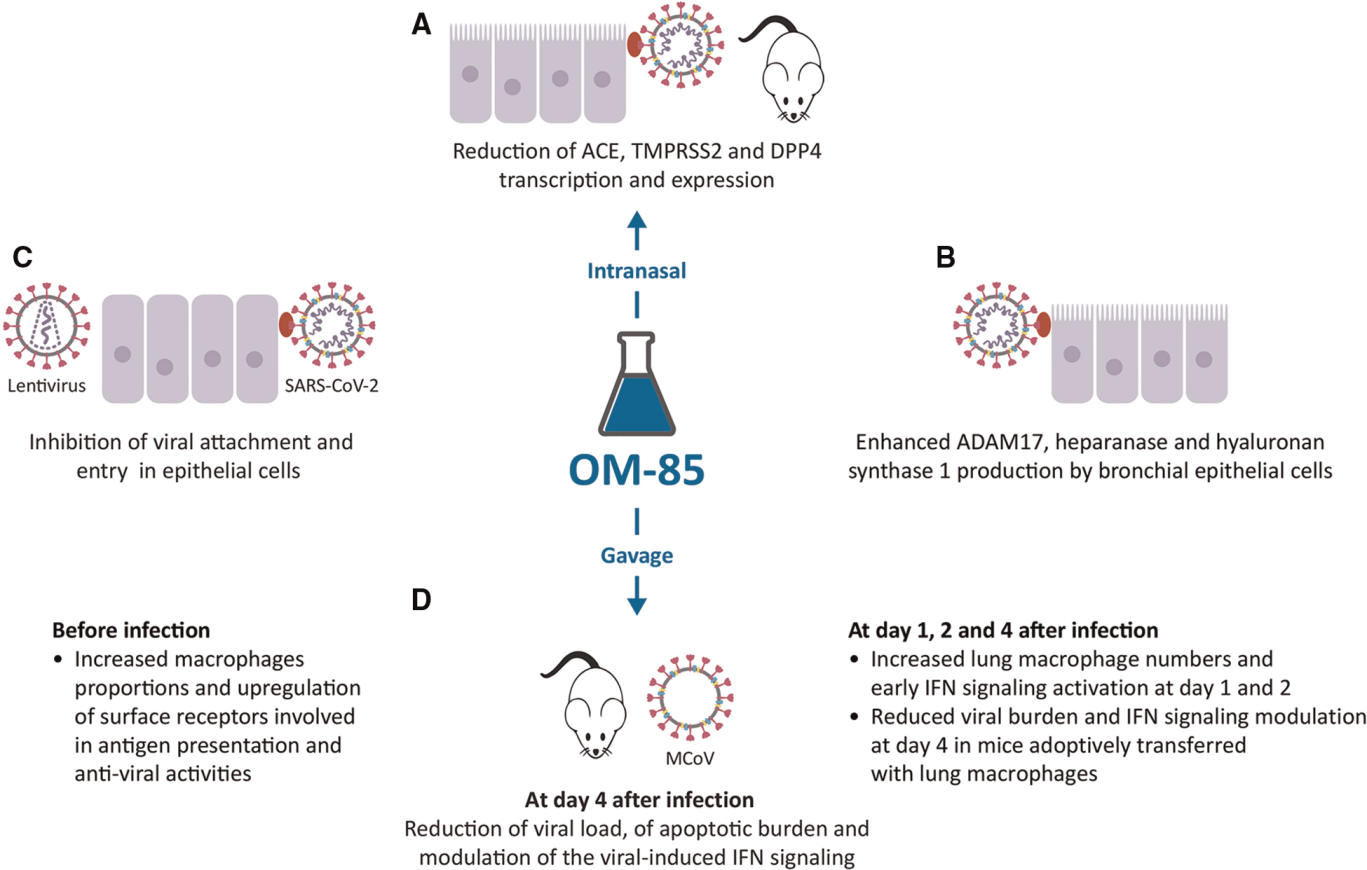
Inhibitory activity of OM-85 on coronavirus infections. (**A**). OM-85-induced inhibition of the transcription of ACE2, TMPRSS2 and DPP4, all proteins involved in Severe Acute Respiratory Syndrome-Coronavirus-2 (SARS-CoV-2) adhesion and infection in mice treated intranasally, and in epithelial cell cultures. (**B**). Increased synthesis of ADAM17, heparanase and hyaluronan synthase 1 by BECs. (**C**). Inhibition of SARS-CoV-2–pseudotyped particles, SARS-CoV-2 attachment, and entry in epithelial cells. (**D**). Effects of OM-85 administered by gavage in SPF mice: increase in macrophage proportions and early IFN signaling activation, reduction of viral load and apoptotic burden, modulation of the viral-induced IFN signaling leading to subsequent reduction of viral load.

SARS-CoV-2 targets human cells through the receptor-binding domain of the structural Spike (S) protein specifically recognizing angiotensin-converting enzyme type 2 (ACE2) receptor protein (46, 47). Following binding to ACE2 receptor, proteolytic cleavage between the S1 and S2 subunits by the transmembrane protease serine subtype 2 (TMPRSS2) is required to facilitate the fusion of the viral and the host cellular membranes and for viral entry (46, 47). Studies in mice, cells derived from SARS-CoV-2 target organs and human epithelial cells, have demonstrated that exposure to OM-85 effectively inhibits the transcription of ACE2 and TMPRSS2 proteins (Figure 3A). In mice, treated intranasally with OM-85 (1 mg/treatment for 14 treatments, over 32 days) ACE2 and TMPRSS2 transcription was significantly inhibited at day 2, and day 2 and 7 respectively, after the last OM-85 treatment (48). Since OM-85 ability to modulate the functions of dendritic cells, depends on Myd88/Trif innate immune signaling (25), this pathway involvement was assessed in wild-type and *Myd88^−/−^Trif^−/−^* C57BL/6 mice. As few as four IN OM-85 treatments were sufficient to reduce by more than half ACE2 and TMPRSS2 transcription in cells isolated from the lungs of wild-type mice, whilst negligible changes were detected in *Myd88^−/−^Trif^−/−^* mice. Also, in Vero E6 cell, Calu-3 cell and Caco-2 cell line cultures, OM-85 (0.48 mg/ml) significantly inhibited ACE2 and TMPRSS2 transcription (48). These results were validated in human BEC lines (BEAS-2B and Nuli) and on primary human BECs, isolated from a healthy individual (49). After exposure for 24 and 48 h to OM-85 (1:50 dilution, in phosphate-buffered saline (PBS), significant inhibition in ACE2 and TMPRSS2 transcription was detected. In kinetic studies, reduction in ACE2 protein expression was shown at all time points (24,48,72 and 96 h), while the downregulating effects on TMPRSS2 protein expression became significant only at day 4 (49).

#### Modulation of the expression of other BEC surface proteins involved in SARS-CoV-2 infection

In addition to ACE2 and TMPRSS2, other molecules expressed on the epithelial cell membrane are involved in SARS-CoV-2 infection. Therefore, the modulation of DPP4*,* ADAM17, heparanase and hyaluronan synthase 1 (Has-1) expression was evaluated, again in BEAS-2B, Nuli BEC lines, and primary human BECs (Figure 3B) (49). DPP4 is a serine exopeptidase expressed in several tissues, which can act as functional receptor by SARS-CoV-2 (50, 51) in experiments performed again in BEAS-2B and Nuli, and primary human BECs, daily treatment with OM-85 (1:50) significantly reduced the levels of DPP4 mRNA and DPP4 protein (i.e., transcription and expression) (49). In contrast with what was observed for ACE2, TMPRSS2 and DPP4, daily treatment with OM-85 significantly increased ADAM17 mRNA and protein levels at all time points (49). ADAM17 (a disintegrin and metalloproteinase domain 17) is engaged in the cleavage and release of ACE2 (52) and its upregulation was associate with an increase extracellular release of ACE2 in its soluble form (sACE2). Circulating sACE2 can bind to the SARS-CoV-2 virus effectively, acting as soluble decoy and blocking attachment to the membrane-bound receptor and virus entry (53, 54). An increase in heparanase secretion and of Has-1, was also demonstrated after OM-85 treatment (respectively at 1:50, and 1:100 or at 1:50 dilutions) (49). Heparanase is an enzyme that degrades polymeric heparan sulfate (HS) produced by epithelial cells (55). Expressed on the cell surface, HS serves as receptor for SARS-CoV-2 and facilitates the interaction between the S protein and its homing receptor ACE2 (56, 57). The upregulation of heparanase secretion was associated with a decrease of HS expression on the cell membrane, i.e., with a decrease of the SARS-CoV-2 binding sites availability. Finally, the increased expression of Has-1 in the presence of OM-85 was associated with an upregulation of the synthesis and secretion of hyaluronic acid (HA). HA is a non-sulphated glycosaminoglycan distributed throughout connective, epithelial and neural tissues (58). Being one of the chief components of the extracellular matrix, HA participates in a variety of processes, including the response to bacterial, fungal, and viral infections (59) and its antiviral and virucidal activity shown *in vitro* could be useful in fighting SARS-CoV-2 (60, 61).

#### Inhibition of virus attachment and cell entry

To investigate whether OM-85 could reduce SARS-CoV-2 S protein attachment to host cells, a recombinant histidine (His)-tagged S1 subunit comprising the SARS-CoV-2 receptor binding domain, was firstly used (48). Incubation with OM-85 (0.48 or 1.92 mg/ml) induced a dose-dependently significant reduction in S1 binding to Vero E6 and Calu-3 cells, but also to HEK293T stably transfected with human ACE2 (ACE2/HEK293T) (48) (Figure 3C). Moreover, OM-85 pretreatment (0.48 mg/ml) strongly inhibited the transduction by SARS-CoV-2–pseudotyped particles in Vero E6 cell but not in ACE2/HEK293T cells, because these ones do not downregulate ACE2 on OM-85 stimulation (48, 49). Therefore, the key event characterizing the OM-85–induced suppression of SARS-CoV-2 infection is the reduction of ACE2 expression. Finally, OM-85 (0.24–1.92 mg/ml) also suppressed Vero cells and Calu-3 cells infection by live SARS-CoV-2 (isolate USA-WA1/ 2020) (48).

### Murine coronavirus (MCoV) infection

To study the cellular processes and the beneficial effects of OM-85 on viral infection and clearance as well as disease outcome a murine coronavirus (MCoV) model was designed by Salzmann M, and coworkers (62).

#### Promotion of lung macrophage anti-viral functions, reduction of viral and apoptotic burden and modulation of IFN signaling on days 4 and 10 after MCoV infection

In SPF mice receiving 7.2 mg OM-85 for 10 consecutive days by gavage before MCoV infection, a massive reduction of viral load was associated with enhanced viral clearance and downregulation of the apoptotic burden in lung tissue on days 4 and 10 (Figure 3D) (62). Strong activation of genes specifically linked to type 1 IFN signaling and innate immunity was observed but in OM-85-treated animals, the reduced viral stress starting at day 4, was associated with a significant downregulation of IFN-α, IFN-*β*, IFN-*γ*, and induced protein with tetratricopeptide repeats 1 (IFIT1) mRNA levels in lung tissue (62). RNA sequencing also indicated differences in antigen processing and presentation, and macrophage activation between treated and untreated mice. Pre-activation of the immune system by OM-85 did not magnify MCoV-induced injury because no adverse effects on lung pathology were detectable-.

#### Early increase in lung macrophage numbers, early IFN signaling activation before and reduced viral burden

Increase in CD11c positive interstitial lung macrophages before virus infection was shown, still retained 2 days after virus infection and associated with overexpression of mRNA levels of genes related to antigen presentation (MHC-II and CD86) and to C1q receptor already before exposure to the virus (62). In the first 2 days of the infection early interferon signaling was present, leading to increased IRF1, IFN-α and IFN-*β* expression in lung tissue and, at day 1, a significant increase was detected of the expression of the retinoic acid-inducible gene 1 (RIG-1), an intracellular receptor involved in the identification of RNA viruses (63). Moreover, mice receiving a transfer of monocytes and macrophages isolated from naive lung tissues before MCoV infection, showed, at day 4, a reduction in lung viral burden and in IFN-α, IFN-*β* and IFIT1 mRNA levels, effects like those induced by OM-85 treatment. Finally, early inhibition of IFN signaling with anti-IFNAR1 antibodies during MCoV infection, after OM-85 treatment, increased viral loads and persistent activation of IFN-related pathways at day 4 post-infection. Therefore OM-85 induced an early “innate immunity training”, leading to lung macrophage pre-activation, to enhanced and faster activation of IFN pathway and its downstream targets allowing for a faster viral clearance together with reduced tissue damage (62).

## Discussion

Many studies and several reviews and meta-analyses have shown the clinical efficacy of OM-85 in the prevention of rRTIs in children (64–68), and in vulnerable adult populations (69–73, 74). More specifically, following oral administration of OM-85, reduction of infection incidence, duration, and severity as well as decreased antibiotic and drug courses have been reported in randomized clinical trials. In addition, this product has shown to reduce the risk for complications in children such as recurrent infectious wheezing episodes or asthma exacerbations (75). In adults with other chronic respiratory disease (e.g., chronic bronchitis, and COPD), OM-85 reduced the exacerbations triggered by respiratory infections (69, 74).

**Figure 4:**
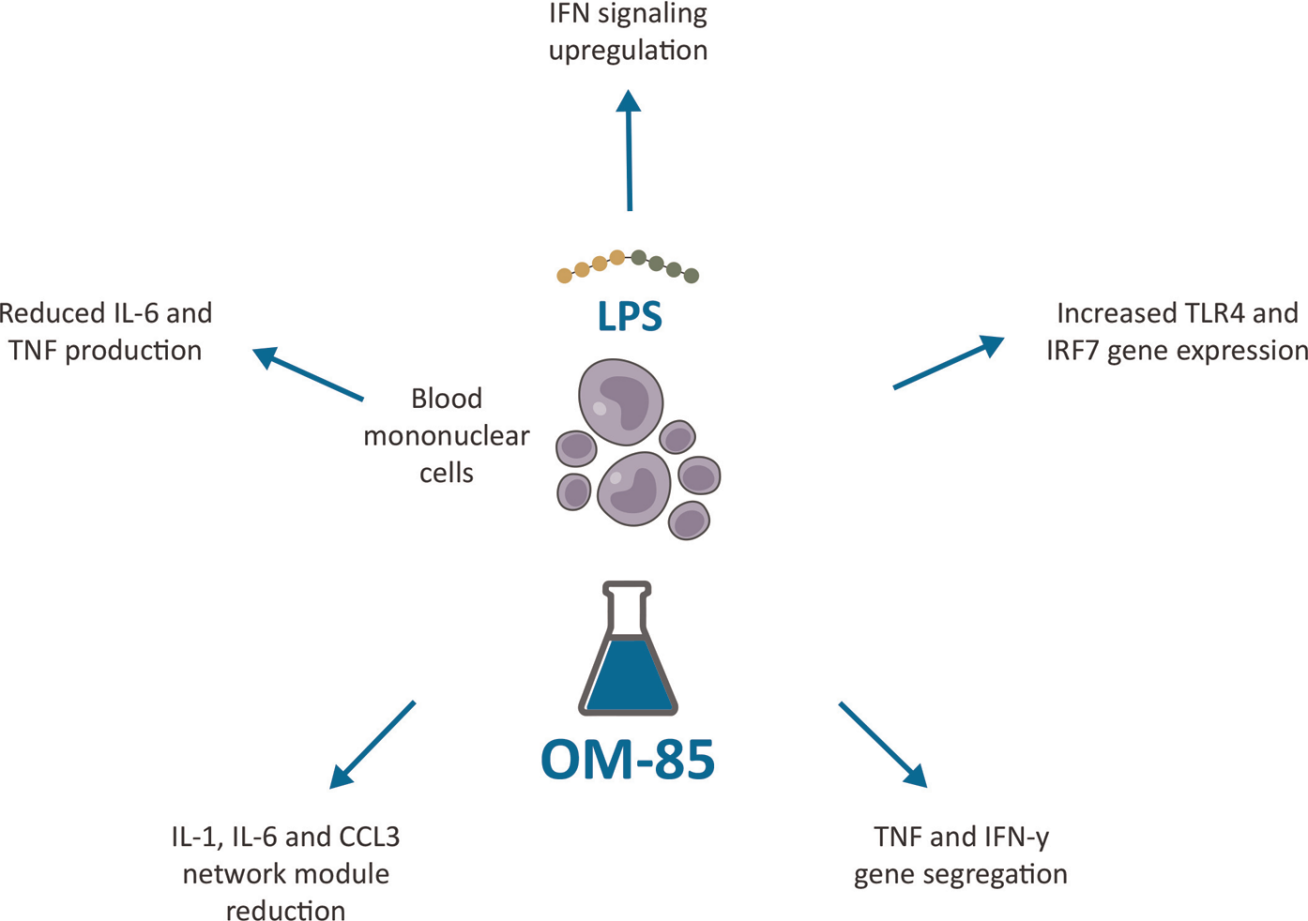
Potential immune training-based effects induced by OM-85 leading to protection against severe lower respiratory tract infections in children. LPS, Lipopolysaccaride; TLR 4, Toll Like Receptor; IRF1, Interferon Regulatory Factor 1.

The underlying mechanisms for the potential immune training-based effects leading to protection against severe lower respiratory tract infections, are only partially understood. However, interesting new data came from a recent placebo-controlled trial in infants, showing that the OM-85-induced reduction in respiratory infection frequency and/or duration, was associated with immune changes indicative of innate immune training (76). Similar changes and effects have been described in the animal model studies included in the present review (39, 62). In the study by Troy and colleagues, human peripheral blood mononuclear cells obtained from infants, were exposed *ex-vivo* to OM-85 (or placebo) and then stimulated with the bacteria mimicking lipopolysaccharide (LPS) (Figure 4) (76). In cultures exposed to OM-85, upregulation of IFN signaling, was accompanied by, (i) network rewiring resulting in increased coordination of TLR4 expression with IFN pathway–associated genes (especially master regulator IRF7); ii) segregation of TNF and IFN-*γ* (which potentially synergize to exaggerate inflammatory sequelae) into separate expression modules; iii) reduced size/complexity of the main proinflammatory network module, containing, IL-1, IL-6, and CCL3. Finally, a reduced capacity for LPS-induced inflammatory cytokine IL-6 and TNF production in the OM-85 group was observed (76). Thus, immune training was associated with and attenuation of potentially pathogenic inflammation. The rationale behind the use of the oral route to administer immunomodulators for the prevention of respiratory conditions centers on the concept of the gut-lung immune axis (15–18). To modulate the inflammatory and immune-mediated responses, gut cells assimilate information directly from the local microorganisms and from the metabolites (e.g., short chain fatty acids) and cytokines they release. This modulation shapes immune responses locally but also at distal organs. Bacterial-derived products (PAMPs/antigens) and metabolites are processed by the gut DCs, which promote T cells and immunoglobulin-producing B cells differentiation and proliferation. Following immune challenges in the respiratory tract these cells, can move into the bloodstream, and traffic from the gut- associated lymphoid tissue (GALT) to the bronchial-associated lymphoid tissue (BALT) and to the airways, where they promote and modulate protective and anti-inflammatory responses (14, 15–18). BLs, such as OM-85, can promote immune homeostasis and anti-viral responses in patients with respiratory infections potentially acting on gut microbiota (77). Consistently, three studies summarized in this review showed the efficacy against IFV and MCoV infection of OM-85 administered to mice by gavage (23, 36, 62). In the first two studies, following administration of 7.2 mg of OM-85-active principle, for 10 days, an increase of influenza-specific NP-tetramer^+^ CD8^+^ cytotoxic T-cell proportions in BAL fluid were associated with a reduced viral replication and a faster viral clearance (23, 36). In the third study, a murine coronavirus (MCoV) model, orally gavage before viral infection with 7.2 mg OM-85 for 10 consecutive days, increased macrophage proportions and upregulated the expression of surface receptors involved in antigen presentation and anti-viral activities. An early IFN signaling activation was associated with the reduction of viral load and with modulation of a strong viral-induced IFN signaling in the “early convalescent” phase (62). In addition, four other studies reported the direct activity of low concentrations of OM-85 on airway epithelial cells (31, 39, 48, 49). In the first study, pre-incubation with OM-85 (10 μg/ml) of primary human BEC cultures infected with HRV, increased the expression of the two anti-viral proteins *β*-defensin and C1qR and the secretion of IFN-*γ* (31) and, at the same time, downregulated the expression of the ICAM-1. Significant inhibition of HRV replication through the cAMP and Erk1/2 MAP kinase pathway activation was detected (31). In the second study, pre-treatment with OM-85 (7.5 µg/ml) of human pulmonary epithelial cell cultures infected with RSV, increased IFN-*β* and ISG15 expression, decreased cell death and reduced RSV-RNA expression (39). In the third and fourth studies, preincubation of human bronchial epithelial cell lines with OM-85 (0.12, 0.24, 0.48, 0.96, or 1.92 mg/ml) modulated the expression of molecules involved in SARS-CoV-2 infection and inhibited virus attachment and cell entry (48, 49). The possibility that these direct effects on airway cells could be translated *in vivo* was addressed in the two studies that evaluated the efficacy of IN administration of OM-85 in mice infected with RSV and SARS-CoV-2 (39, 48). In the first study, IN administration of OM-85 (1 mg/treatment) for 5 days prior to RSV infection in BALB/c mice inhibited viral replication and reduced perivascular and peri bronchial inflammation in the lungs (39). The reduced viral load was associated with increased proportions of alveolar macrophages and a selective set of tolerogenic DCs, and with the expansion of Treg and Th1 lymphocyte expansion in the lung, contributing to a better Th1/Th2 lymphocyte balance and preventing ILC2 recruitment in the airways. The antiviral response was improved by the increased expression of IFN-*β* and of ISG15 in the lung. In the second study, IN treatment of mice with OM-85 (1 mg/treatment for 14 treatments, over 32 days) significantly inhibited ACE2 and TMPRSS2 transcription, at day 2 and at day 2 and 7 after the last OM-85 treatment, an activity that was Myd88/Trif innate immune signaling-dependent (48). Interestingly, a similar protocol involving OM-85 IN administration was used in mice asthma models (41). OM-85 administration (1 mg/treatment every 2 to 3 days, 14 times total), suppressed allergic asthma in different strains of mice, sensitized and challenged with ovalbumin or *Alternaria*. Among the multiple components of the innate and adaptive immune response targeted by OM-85 in that asthma study, preeminent was the epithelium/IL-33/ILC2 axis that is known to initiate type 2 inflammation in the airway mucosa also in RSV infection (78), and the CD103^+^CDs, whose *Myd88/ Trif*-dependent tolerogenic reprogramming was sufficient to transfer OM-85-induced asthma protection (41). Similarly, also in the RSV study the tolerogenic CD103^+^ DC population was significantly increased in OM-85 and in OM-85 + RSV-infected mice, and their expansion was and associated with the expansion of FoxP3^+^ Treg cells and Th1 lymphocytes in the lung, contributing to a better Th1/Th2 balance and to a prevention ILC2 recruitment in the airways (39). In an asthmatic mice model, established with OVA challenge, OM-85 increased the lung FoxP3^+^ Treg cell numbers, reduced the BAL eosinophil numbers, and downregulated both the IgE and Th2 pro-inflammatory cytokine production (79). Studies in children with rRTI have shown that OM–85 promotes immune system maturation *via* downregulation of Th2 lymphocyte activity, through the activation of FoxP3^+^ Treg cells, which are important for the immune response regulation and for viral clearance (17). Notably, in the two studies, OM-85 administration through the IN route resulted into significant inhibition of RSV replication and an almost complete asthma suppression at cumulative doses several fold lower than those reportedly used through the oral route.

## Conclusions

As shown in pre-clinical research, OM-85 can promote an effective anti-viral activity, by modulating the structure and the functions of a large numbers of airways epithelial and immune cells, when administered orally, but also intranasally, as shown in several mice experiments. In addition, in line with the hygiene hypothesis and innate trained immunity concepts, it has been observed that oral bacterial-derived products might mimic the microbiota functions. There is some preliminary evidence that the product might also stabilize or rearrange the microbiota to promote a balanced immune state and respiratory health, but more data is needed. The positive effect of IN-administered OM-85 in regulating the response to infections and in asthma prevention, may reflect both the microbial stimulation that the BL provides at epithelial layer level and its direct access to the cellular and molecular networks that reside in the airway mucosa. The results of these studies which demonstrate that OM-85 can directly enhance anti-viral capacity through the engagement of a variety of cells, suggest that the administration of OM-85 in the airways could be a valuable option in clinical practice, a hypothesis that needs to be confirmed by future research and clinical trials.
